# Supplementary material for: Mechanic Insight into the Distinct and Common Roles of Ovariectomy Versus Adrenalectomy on Adipose Tissue Remodeling in Female Mice
Source: Int J Mol Sci. 2023 Jan 24;24(3):2308. doi: 10.3390/ijms24032308 (PMC9916485; doi:10.3390/ijms24032308)
Supplement: Supplementary file 1 [file ijms-24-02308-s001.zip › Supplemental Materials.pdf]

Supplementary Materials

Mechanic insight into the distinct and common roles of ovariectomy versus adrenalectomy on adipose tissue remodeling in female mice

Weihao Chen, Fengyan Meng, Xianyin Zeng, Xiaohan Cao, Guixian Bu, XiaoGang Du, Guozhi Yu, Fanli Kong, Yunkun Li, Tian Gan and Xingfa Han*

Isotope Research Lab, Biological Engineering and Application Biology Department, Sichuan Agricultural University, Ya'an 625014, China
* Correspondence: Xingfa Han (xfhan2012@sicau.edu.cn)

Supplementary figures:

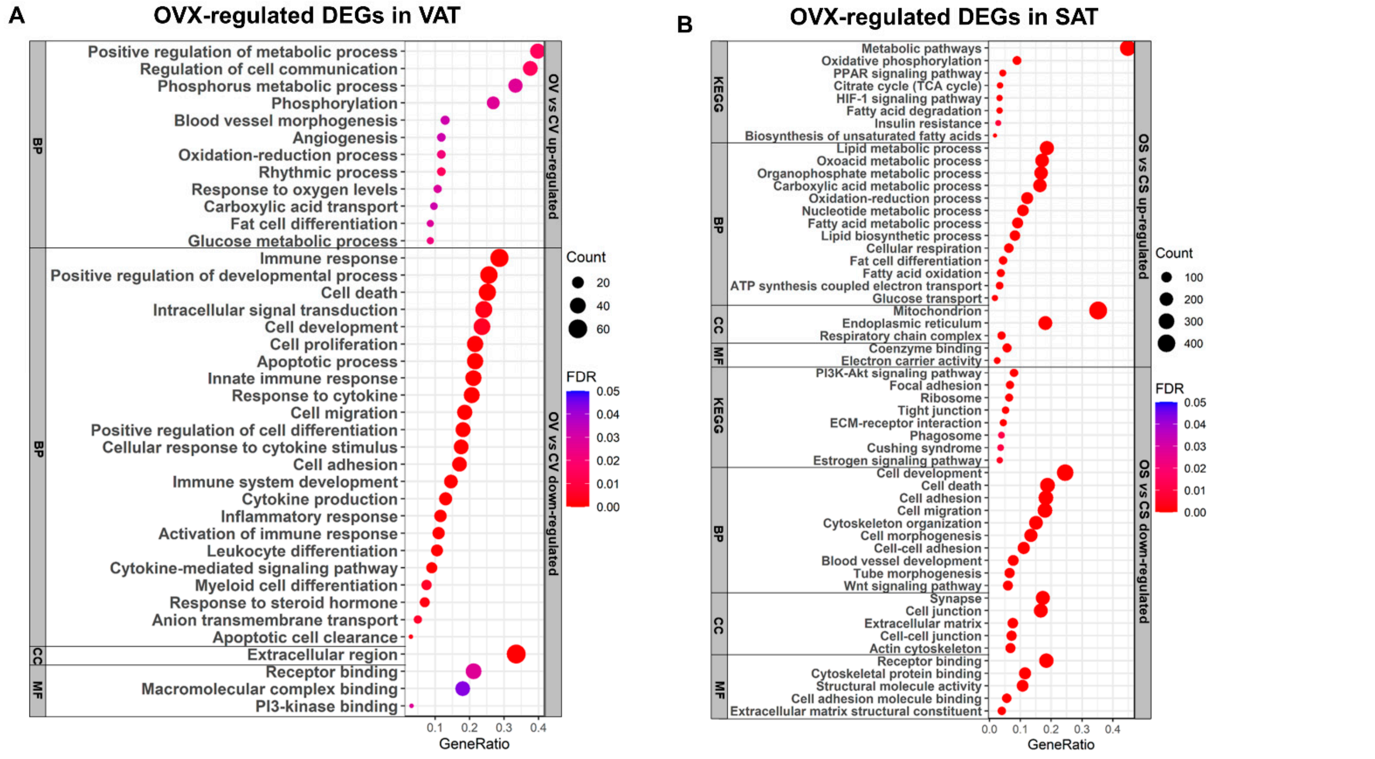

Figure S1. Functional enrichment analysis of DEGs in VAT/SAT between OVX and sham control mice. **(A)** Functional enrichment analysis of the up-regulated/down-regulated DEGs in VAT between OVX and sham controlmice. **(B)** Functional enrichment analysis of the up-regulated/down-regulated DEGs in SAT between OVX and sham controlmice. KEGG, Kyoto encyclopedia of genes and genomes; BP, Biological process; CC, Cellular component; MF, Molecular function.

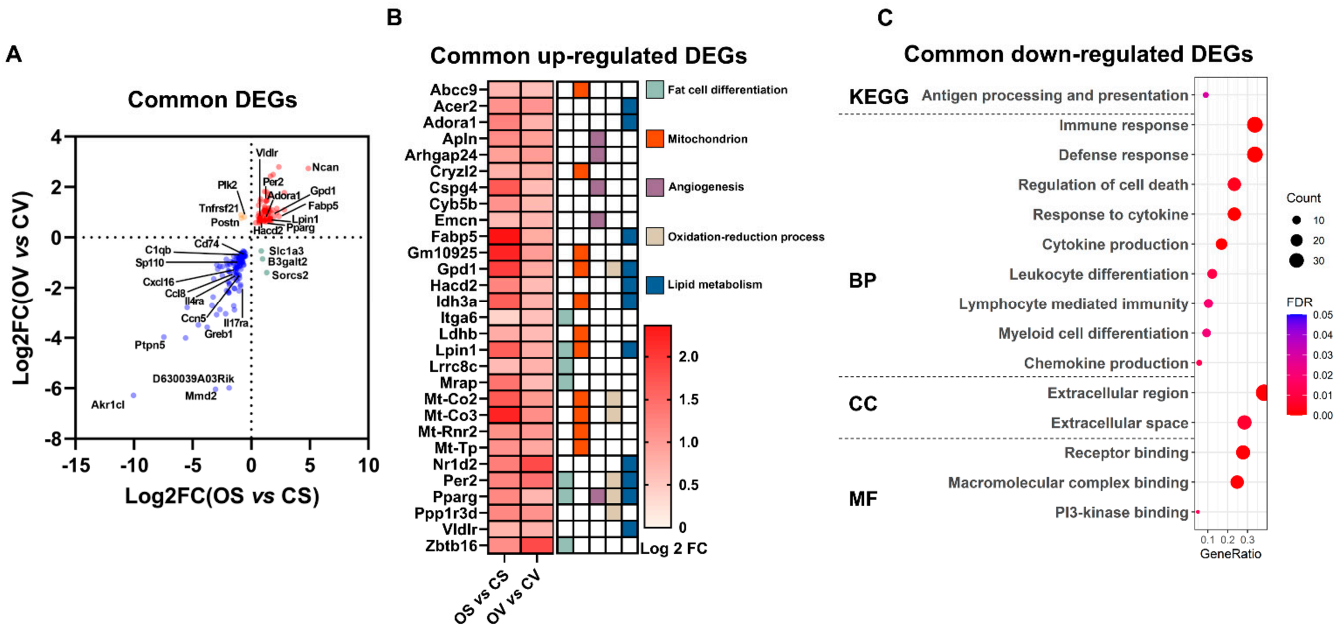

Figure S2. Common functional changes of VAT and SAT in response to OVX. **(A)** The common regulated genes in both SAT and VAT by OVX. **(B)** Heatmap of the metabolism-associated common up-regulated DEGs in both SAT and VAT by OVX. **(C)** Functional enrichment analysis of the common down-regulated DEGs in both SAT and VAT by OVX.

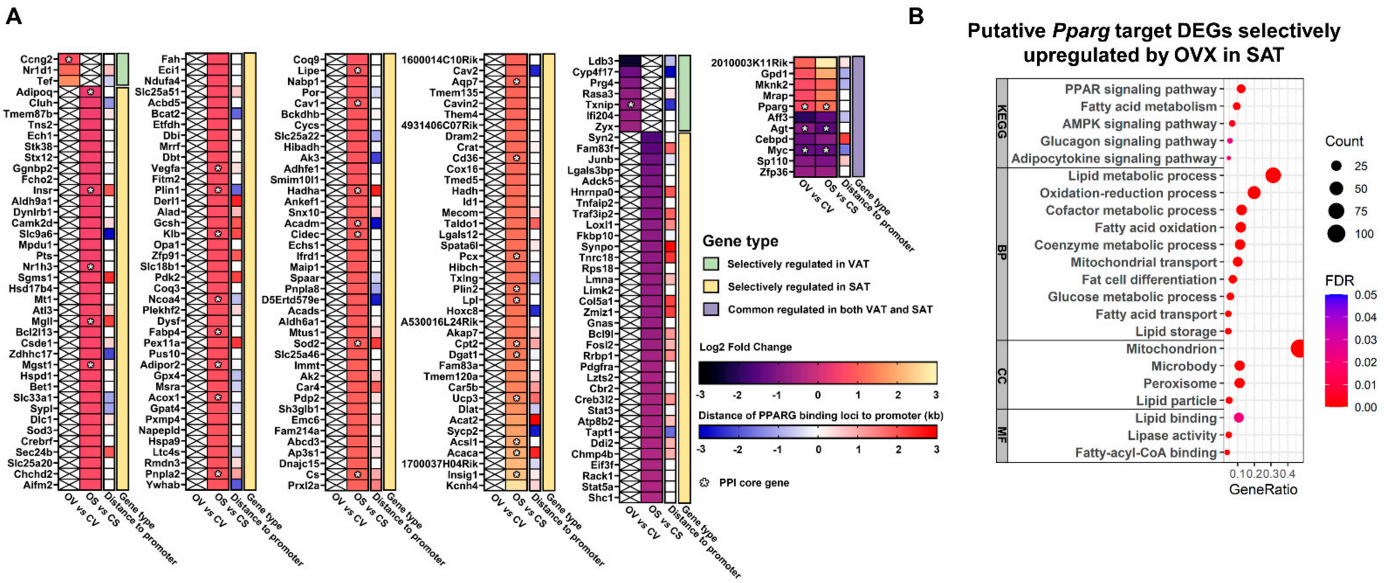

Figure S3. Putative *Pparg* target DEGs regulated by OVX in VAT and SAT. **(A)** Heatmap of all putative *Pparg* target DEGs regulated by OVX in SAT and VAT. **(B)** Functional enrichment analysis of putative *Pparg* target genes selectively upregulated by OVX in SAT.

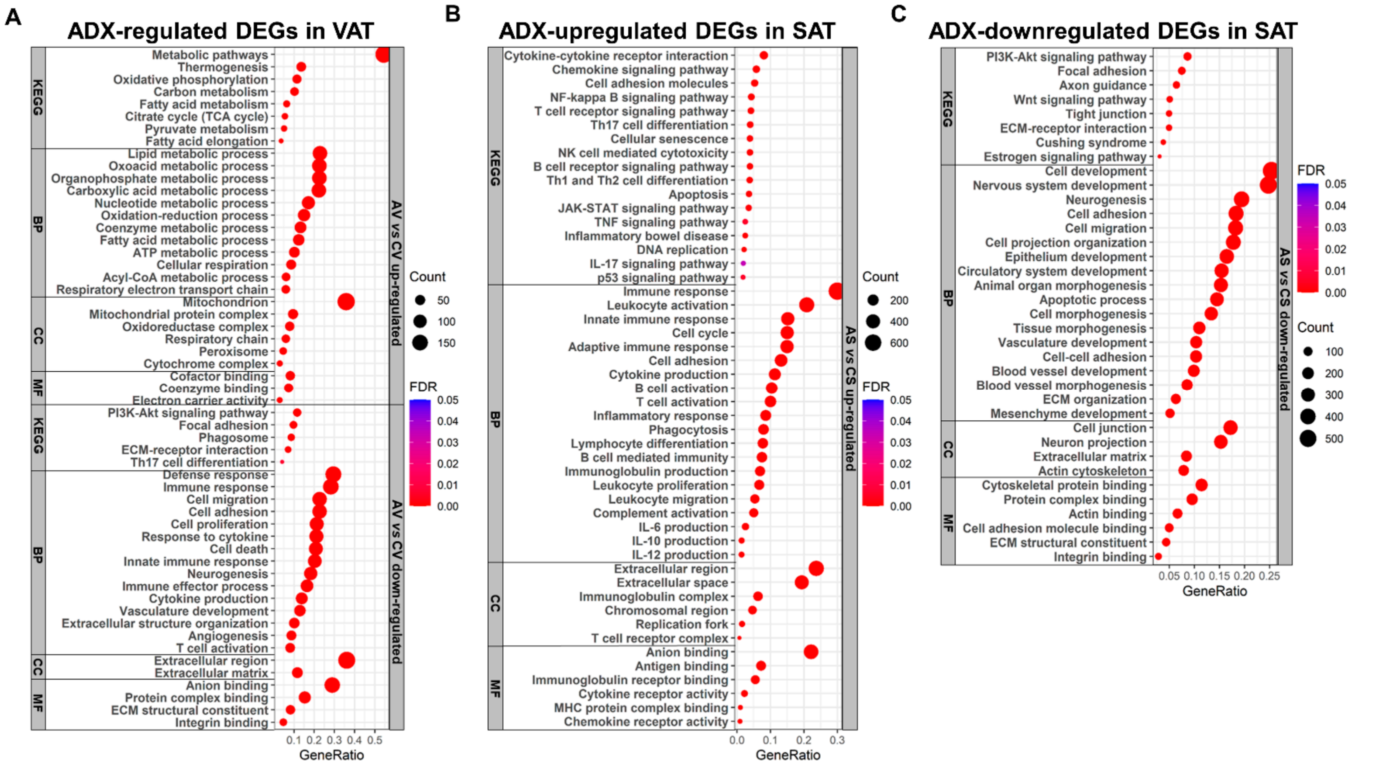

Figure S4. Functional enrichment analysis of DEGs in VAT/SAT between ADX and sham control mice. **(A)** Functional enrichment analysis of the up-regulated and down-regulated DEGs between

VAT of ADX and sham control mice. **(B)** Functional enrichment analysis of the up-regulated DEGs between SAT of ADX and sham control mice. **(C)** Functional enrichment analysis of the down-regulated DEGs betweenSAT of ADX and sham control mice.

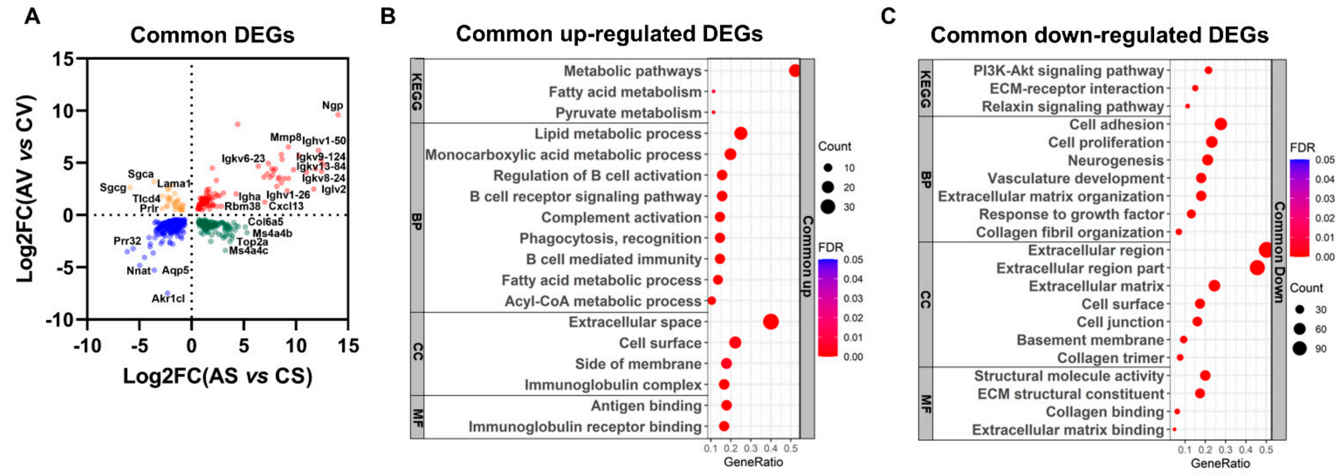

Figure S5. Common functional changes of VAT and SAT in response to ADX. **(A)** The common regulated genes by ADX in both SAT and VAT. **(B)** Functional enrichment analysis of the common up-regulated DEGs by ADX. **(C)** Functional enrichment analysis of the common down-regulated DEGs by ADX.

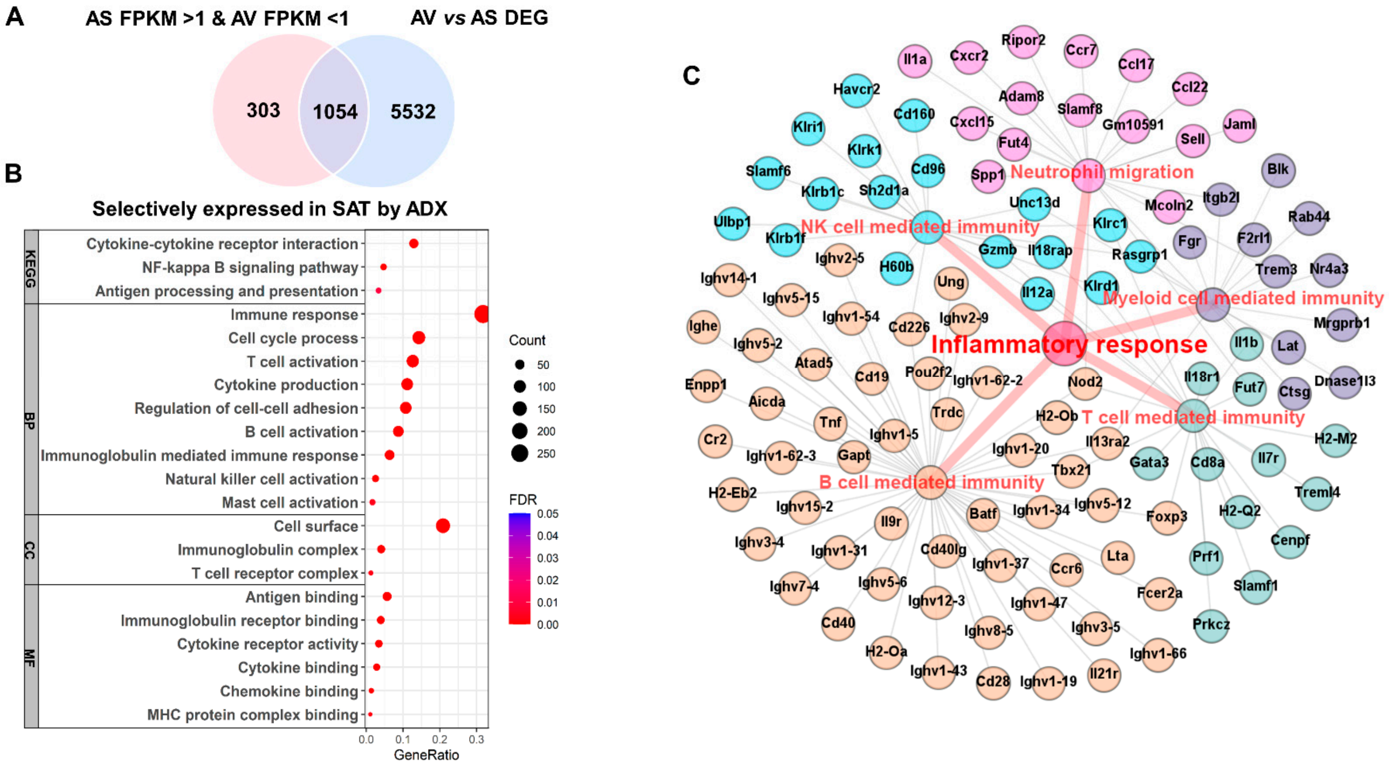

Figure S6. SAT selectively expressed DEGs in ADX mice were mainly involved in immune response. **(A)** Venn diagram of SAT selectivily expressed DEGs in ADX mice. **(B)** Functional enrichment analysis of 1054 SAT selectively expressed DEGs in ADX mice. **(C)** A network map indicated SAT selectively expressed DEGs in ADX mice were mainly involved in inflammatory response mediated by a variety type of immune cells.

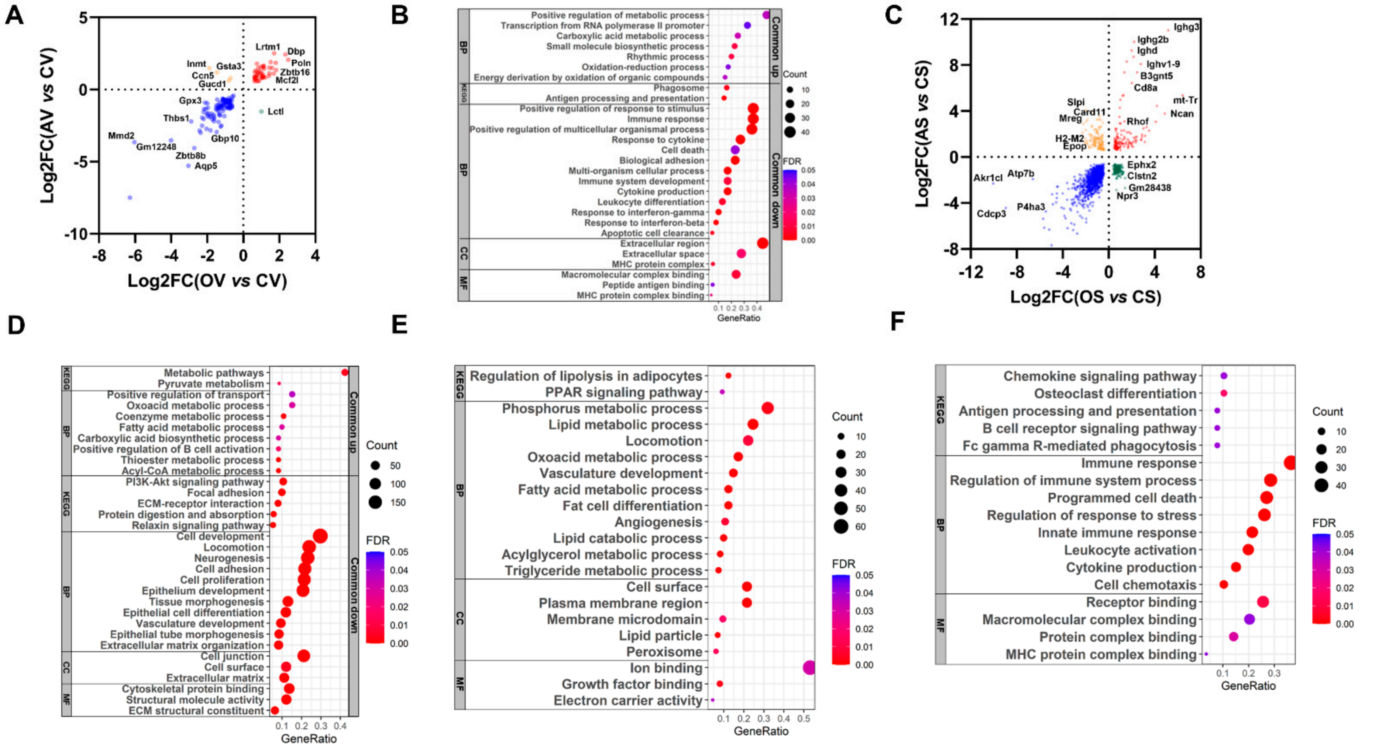

Figure S7. The common regulated genes in VAT/SAT by both OVX and ADX. **(A)** The common regulated genes in VAT by both OVX and ADX. **(B)** Functional enrichment analysis of the commonly and consistently regulated genes in VAT by both OVX and ADX. **(C)** The common regulated genes in SAT by both OVX and ADX. **(D)** Functional analysis of the commonly and consistently regulated genes in SAT by both OVX and ADX. **(E)** Functional analysis of the DEGs up-regulated by OVX but downregulated by ADX in SAT. **(F)** Functional analysis of the DEGs down-regulated by OVX but upregulated by ADX in SAT.

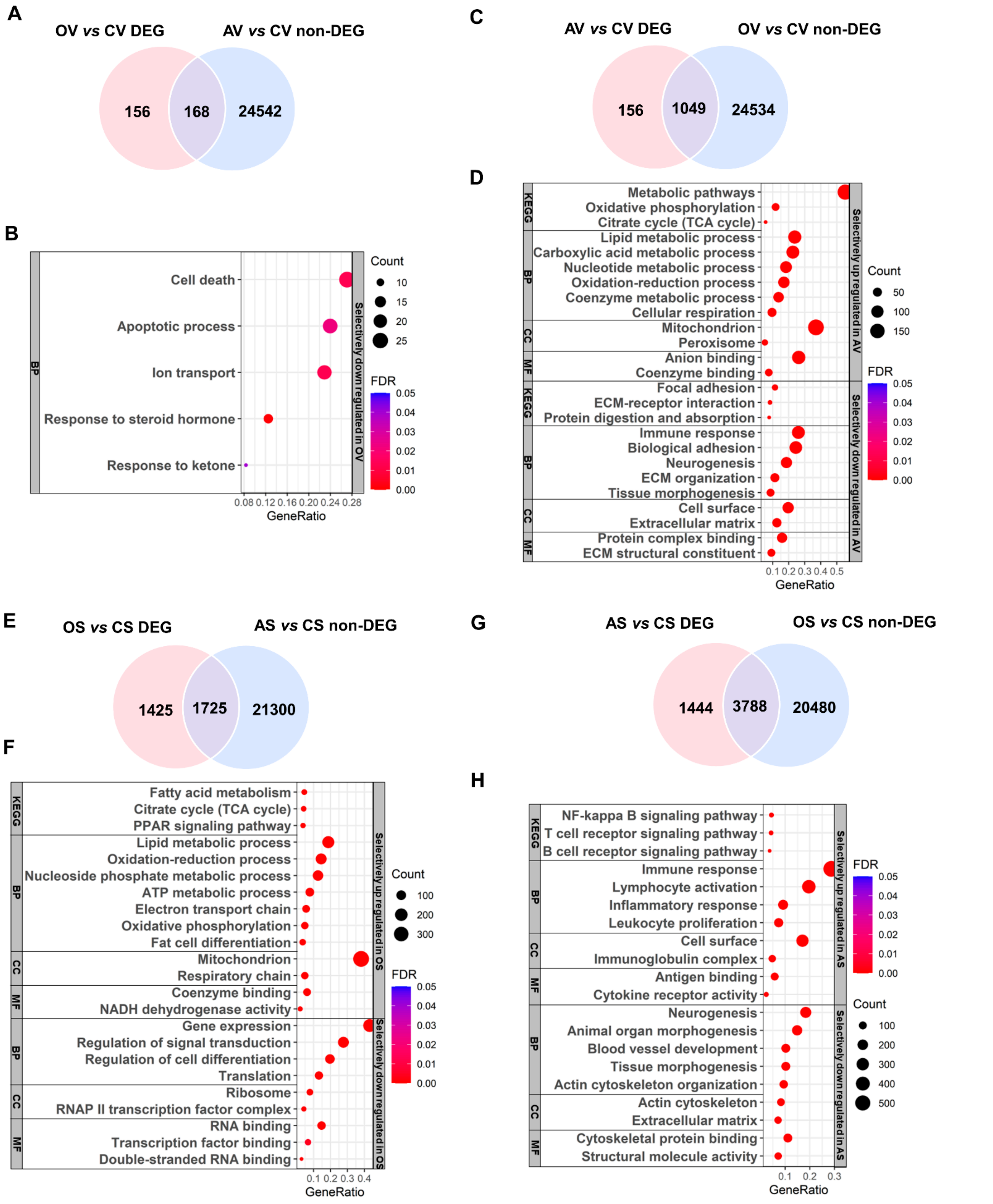

Figure S8. Distinct responses induced by OVX or ADX. **(A)** Venn diagram of distinct DEGs in VAT by OVX. **(B)** Functional enrichment analysis of the distinct DEGs in VAT by OVX. **(C)** Venn diagram of distinct DEGs in VAT by ADX. **(D)** Functional analysis of the distinct DEGs in VAT by ADX. **(E)** Venn diagram of distinct DEGs in SAT by OVX. **(F)** Functional analysis of the distinct DEGs in SAT

by OVX. **(G)** Venn diagram of distinct DEGs in SAT by ADX. **(H)** Functional analysis of the distinct DEGs in SAT by OVX.
