# Supplementary material for: Mechanic Insight into the Distinct and Common Roles of Ovariectomy Versus Adrenalectomy on Adipose Tissue Remodeling in Female Mice
Source: Int J Mol Sci. 2023 Jan 24;24(3):2308. doi: 10.3390/ijms24032308 (PMC9916485; doi:10.3390/ijms24032308)
Supplement: Supplementary file 1 [file ijms-24-02308-s001.zip › Supplemental tables.pdf]

Supplementary tables:

Tabel S1 Differentially expressed genes (DEGs) in pairwise groups;

Tabel S2 Functional enrichment analysis of DEGs using DAVID;

Table S3 Common regulated DEGs by both OVX and ADX;

Tabel S4 Functional enrichment analysis of common DEGs using DAVID;

Table S5 SAT selectivily expressed DEGs under ADX treatment;

Table S6 Distinct DEGs induced by OVX or ADX;

Table S7 Functional enrichment analysis of the distinct DEGs induced by OVX or ADX using DAVID.
